# The Mandibular and Hyoid Arches—From Molecular Patterning to Shaping Bone and Cartilage

**DOI:** 10.3390/ijms22147529

**Published:** 2021-07-14

**Authors:** Jaroslav Fabik, Viktorie Psutkova, Ondrej Machon

**Affiliations:** 1 Department of Developmental Biology, Institute of Experimental Medicine of the Czech Academy of Sciences, 14220 Prague, Czech Republic; jaroslav.fabik@iem.cas.cz (J.F.); viktorie.psutkova@iem.cas.cz (V.P.); 2 Department of Cell Biology, Faculty of Science, Charles University, 12800 Prague, Czech Republic

**Keywords:** neural crest cells, craniofacial development, pharyngeal arches, jaw development, hyoid bone, patterning, cartilage, bone, chondrogenesis, osteogenesis

## Abstract

The mandibular and hyoid arches collectively make up the facial skeleton, also known as the viscerocranium. Although all three germ layers come together to assemble the pharyngeal arches, the majority of tissue within viscerocranial skeletal components differentiates from the neural crest. Since nearly one third of all birth defects in humans affect the craniofacial region, it is important to understand how signalling pathways and transcription factors govern the embryogenesis and skeletogenesis of the viscerocranium. This review focuses on mouse and zebrafish models of craniofacial development. We highlight gene regulatory networks directing the patterning and osteochondrogenesis of the mandibular and hyoid arches that are actually conserved among all gnathostomes. The first part of this review describes the anatomy and development of mandibular and hyoid arches in both species. The second part analyses cell signalling and transcription factors that ensure the specificity of individual structures along the anatomical axes. The third part discusses the genes and molecules that control the formation of bone and cartilage within mandibular and hyoid arches and how dysregulation of molecular signalling influences the development of skeletal components of the viscerocranium. In conclusion, we notice that mandibular malformations in humans and mice often co-occur with hyoid malformations and pinpoint the similar molecular machinery controlling the development of mandibular and hyoid arches.

## 1. Introduction

The primary function of the gnathostome facial skeleton is to encase the openings to the mouth and airways and to accommodate several sensory organs (such as vision, smell, or taste). This ancestral function of facial skeleton is shared among all of the gnathostome species and may have played a central role in their evolution. The facial skeleton of gnathostomes, also known as the viscerocranium, is composed of bone and cartilage that collectively form the skeleton of the face and throat. The membranous viscerocranium is formed by a process of intramembranous ossification, whereas the cartilaginous viscerocranium utilizes the endochondral ossification to form bones. Since the last common ancestor of mammals and teleosts roamed the Earth ≈450 million years ago, the composition of murine and zebrafish viscerocrania is vastly different [1]. However, studies show that genetic regulation of craniofacial morphogenesis between the mouse and the zebrafish is similar, indicating a common regulatory circuit during facial development among gnathostomes. This relative similarity means that the zebrafish is complementary to the mouse in the research of craniofacial defects. Currently, the research of zebrafish craniofacial development is growing in intensity, since the genetic machinery controlling PA development is similar among zebrafish, mice, and humans. Some researchers even use a zebrafish model to study human craniofacial diseases, such as CATSHL syndrome (tall stature and hearing loss) and cleft lip/palate [2,3,4]. The adult viscerocranium is composed of many individually distinct elements and requires a coordinated integration of various tissues. Intricate molecular signals and transcription factors among cranial tissues regulate the patterning of the prospective face, which ensures the formation of heterogenous bone and cartilage. Perturbation and impaired regulation of craniofacial development results in dysmorphy of bones and cartilage, which collectively accounts for at least a third of all birth defects in humans [5]. Understanding the precise mechanism of how bony and cartilaginous structures arise and attain their distinct shape may improve treatment and reduce the impact of certain craniofacial birth defects on human patients. During the embryonic development in amniotes, transient embryonic structures known as pharyngeal arches (PAs) undergo extensive growth and differentiation to create the adult viscerocranium. Pharyngeal arches are a series of paired, bilaterally symmetrical outgrowths on both sides of the developing pharynx. Cells from all germ layers take part in assembling the PAs. Each PA consists primarily of two robust mesenchymal populations, the neural crest-derived mesenchyme (also known as “ectomesenchyme”) and the paraxial mesoderm [6]. The oral surface of arches is coated with the ectodermal epithelium, whereas the pharyngeal surface is lined with the endodermal epithelium. The neural crest (NC), sometimes colloquially termed as the fourth germ layer, is a multipotent embryonic population of cells that arises at the lateral border of the neural plate, from which it subsequently delaminates and undergoes extensive migration into distant parts of the body [7]. Neural crest cells (NCCs) are regarded as multipotent because they have the capacity to differentiate into plethora of cell types—osteoblasts, chondroblasts, fibroblasts, neurons, and glia, among many others [8]. A subpopulation of NCCs coming from the level of the future brain—named the cranial neural crest—gives rise to many tissues, including the viscerocranium, the connective tissues, and part of the neurosensory ganglia of the cranium [9,10]. On the other hand, the cranial paraxial mesoderm within PAs forms the muscles and blood vessels of the face, neck, and throat [11].

The segmentation of the pharyngeal region appears to be driven by the endoderm and is independent of NCCs that migrate into PAs [12]. Moreover, the pharyngeal endoderm provides positional clues for the mesenchyme within PAs and is also responsible for the formation of particular arch components. Via interaction with migrating NCCs, the mesoderm actively participates in the formation of PAs [13]. The cranial paraxial mesoderm proliferates ahead of the neural crest migratory front, thus prior to the migration of the neural crest. Like the NC, the mesoderm is inherently motile. Proliferating mesodermal cells commence the PA formation by driving outgrowth in the lateral direction. After the initiation of NC migration, a portion of mesodermal cells freely intermingles with NCCs, while others are displaced by migrating NCCs. After NCCs invade the nascent arches, they actively proliferate in order to stay in pace with the mesoderm. Thus, the mesoderm is the main driver of PA growth, and PAs can form even the absence of NC. The fact that mouse mutants lacking specific NC streams will still form normal PAs supports the notion that NCCs are not required during the initial stages of PA formation. Thus, the formation of the PA template precedes the appearance of NCCs in the pharyngeal region [14,15]. This narrative review focuses on the morphogenesis and skeletogenesis of the first two PAs in mice and zebrafish, since both these models are used in the research of craniofacial diseases. Tight control of temporospatial cell specification and differentiation along the anatomical axes is crucial for the embryonic formation of various structures. The authors present an overview of signalling pathways and regulatory networks involved in this process in the mandibular and hyoid arches. Furthermore, we outline that the mandibular and hyoid arches are collectively governed by a shared gene regulatory network. 

## 2. Anatomy and Fate of Pharyngeal Arches

In amniotes, including humans, there are five PAs, numbered first, second, third, fourth, and fifth. Previously, the terminal arch used to be labelled as the sixth, while the fifth was considered rudimentary, disappearing almost as soon as it has formed. However, new analyses show that there is no evidence from amniote embryology for the existence of a transient, rudimentary fifth arch [16]. Collectively, the abnormal development of PAs is linked to several major groups of birth defects in humans [17]. The first two PAs are called the mandibular and the hyoid and have been named according to the anatomical structures they turn into in the adult organism. The third, fourth, and fifth PAs are collectively known as the posterior pharyngeal arches. After formation of the mandibular arch, the first PA is split into upper maxillary and lower mandibular processes. Cartilage elements and endochondral bone originating from PAs collectively make up the splanchnocranium. In the mandibular arch, two cartilaginous elements arise—a rod shaped, oblongate Meckel’s cartilage in the mandibular process and subtler palatoquadrate cartilage in the maxillary process. During craniofacial morphogenesis, palatoquadrate cartilage undergoes endochondral ossification to form a portion of orbital and lateral skull wall, the alisphenoid, and the second middle ear bone, the incus [18,19,20]. In contrast, a fraction of NCCs encasing the splanchnocranium differentiates directly into functional osteoblasts without a cartilaginous intermediate by the process of intramembranous ossification. In the maxillary process, NCCs surrounding the palatoquadrate cartilage form the maxilla, zygomatic, and squamous part of the temporal bone [21]. Facial bones, which are created around splanchnocranial cartilages, collectively comprise a membranous viscerocranium and serve as the functional jaws in mammals. Interestingly, in the mouse, only NCCs in the first PA have the potential to generate osteoblasts that undergo intramembranous ossification.

In all gnathostomes, Meckel’s cartilage represents a strut of the lower jaw during embryonic development. Meckel’s cartilage initially consists of a pair of continuous rods of cartilage, which subsequently elongate anteriorly and later fuse in the distal midline to form a V-shaped structure outlining the forming lower jaw in mice. In mammals, Meckel’s cartilage can be divided into three parts according to the fate of each region: anterior/distal, intermediate/central, and posterior/proximal [22,23,24,25]. In humans, the distal part of Meckel’s cartilage undergoes endochondral ossification and forms a portion of dentary bone extending from the mental foramen to the midline. However, isolated cartilaginous nodules originating from Meckel’s cartilage can be found on the dorsal surface of the mandibular symphysis [26]. The most proximal part of Meckel’s cartilage turns to bone and forms the first middle ear bone—the malleus. Even though the intermediate part of Meckel’s cartilage initially serves as a template during the development of the lower jaw, it later degenerates, and a dentary bone emerges in its place, also known as the jawbone or the mandible. Although chondrocytes in Meckel’s cartilage have been shown to be able to transdifferentiate into osteogenic cells, evidence for the ossification of the intermediate part of Meckel’s cartilage in vivo is currently limited [27,28,29,30,31,32]. Most importantly, the cartilaginous matrix of Meckel’s cartilage is removed during the mandibular development. Nonetheless, two separate parts of the intermediate region of Meckel’s cartilage, one at the base of skull and the other just at the periphery of the mandibular foramen, ultimately undergo endochondral ossification and turn into the spine of the sphenoid and the lingula of the mandible, respectively. In the adult organism, the dentary bone and the malleus are interconnected by ligaments. Parts of Meckel’s cartilage connecting the spine and lingula are thought to transdifferentiate to become the anterior ligament of the malleus and sphenomandibular ligament [33,34]. The sphenomandibular ligament connects the lingula of the mandible, situated at the periphery of the mandibular foramen and the spine of the sphenoid, hanging from the cranial base, from which it continues as an anterior ligament of the malleus to the middle ear cavity and attaches itself to the malleus. In adulthood, the connection between the mandible and the middle ear is still apparent, as trauma to the jaw joint can potentially cause dislocation of ear bones [35]. After the dentary bone undergoes intramembranous ossification, secondary ossification centres appear in the key points of articulation and mechanical force—in the condylar, coronoid, and angular processes of the mandible—where they initiate endochondral ossification. Since Meckel’s cartilage acts as a template for later formation of the lower jaw bones, its defects lead to anomalies in the pattern and size of the lower jaw in both mouse and human embryos [36]. In summary, Meckel’s cartilage turns into diverse structures along the proximal-distal axis: the malleus; ligaments, replaced by the dentary; and mandibular symphyseal cartilage [25]. 

Generation of bone in the second PA generally involves endochondral ossification [37]. In the mammalian hyoid arch, several separate cartilaginous elements arise, i.e., anlage of the third middle ear bone—the stapes, and Reichert’s cartilage. Unlike Meckel’s cartilage, Reichert’s cartilage is not a continuous structure [38]. The cranial portion of Reichert’s cartilage is continuous with the ear capsule and undergoes endochondral ossification to form a bony projection of the temporal bone, termed the styloid process. The smaller caudal segment of Reichert’s cartilage develops in close relation to the oropharynx and undergoes endochondral ossification to form lesser horns of the hyoid. No cartilage connection between these segments exists, although they are temporarily linked by a mesenchymal band, which is thought to differentiate into muscles and ligaments [39]. The cartilage element of the third PA does not bear any eponymous name and contributes to the development of greater horns of the hyoid and possibly to superior horns of the thyroid [40]. The body of the hyoid bone originates from a single growth centre, without overt contributions from the second PA and third cartilage elements. In mammals, posterior PAs probably bear a miniscule importance. Analyses of chondrogenesis and myogenesis in the chick and mouse, as well as three-dimensional analysis of human embryos, revealed that cartilage formation does not occur within the fourth and fifth PAs [41,42]. Laryngeal cartilages, previously considered to be derived from the posterior PAs, likely develop as new mesenchymal condensations in the throat region [41].

Interestingly, reports of abnormalities in the hyoid arch in humans are uncommon in the literature. However, severe hyoid abnormalities associated with swallowing dysfunction occur in patients with Pierre Robin sequence [43], and infants with cleft lip and palate occasionally exhibit delayed ossification of the hyoid bone, as well as a significantly lower position of the hyoid bone relative to the cervical vertebrae [44]. Conversely, the hyoid bone has been shown to have a more superior and posterior position in patients with hyperdivergent vertical facial growth [45]. In a 15-year-old boy patient with cleidocranial dysplasia, Yoshida et al. reported a unique case of abnormal ossification of the hyoid bone [46]. Cephalometry of children with 22q11.2 deletion syndrome revealed a reduction of hyoid bone lengths, and hyoidal gaps, which reflect the fusion of the hyoidal segments, the greater horns, and the body, were larger than those of the controls [47]. This finding indicates that the ossification of the hyoid bone is delayed in children with 22q11.2 deletion syndrome. In accordance with this, the delayed ossification of the hyoid bone was suggested to be a useful tool in the diagnosis of DiGeorge syndrome during the first postnatal months, before the diagnostic use of the FISH hybridization techniques [48]. Moreover, autopsied infants with DiGeorge syndrome, tetralogy of Fallot, and interrupted aortic arch showed a significantly low incidence of visible hyoid ossification centre [49]. Since the hyoid bone has an important role in respiration, deglutition, and speech, delayed development of the hyoid bone in children with 22q11.2 deletion syndrome may be related to hypotonia of the velopharyngeal muscles and nasal speech. 

In teleosts, seven PAs, numbered first, second, third, fourth, fifth, sixth, and seventh, have been described. As a hallmark of gnathostomes, the first PA/the mandibular arch in teleosts transforms into the jaws during embryogenesis. The second PA, the hyoid arch, mainly provides the attachment of the jaws to the base of neurocranium. The remaining five PAs, also known as branchial/gill arches, provide a gill-supporting function. The mandibular arch is divided into two clearly distinguishable cartilaginous bars, Meckel’s cartilage and palatoquadrate cartilage. While Meckel’s cartilage is the precursor of the lower jaw, dorsally situated palatoquadrate cartilage precedes the appearance of individual bones in the upper jaw. Similar to the mandibular, the hyoid arch is divided into a dorsal region, represented by hyosymplectic cartilage, and a ventral region, represented by ceratohyal and basihyal cartilages. The hyosymplectic cartilage in the dorsal region provides the attachment of jaw to the neurocranium, whereas the ventrally situated ceratohyal and basihyal cartilages act to stabilize the jaw and support the neck region. This primary setting of the teleost viscerocranium represents a base for further development, which is the bone formation. The mechanism of ossification in teleosts slightly differs from that in mammals. At first, endochondral bone usually goes through a perichondral ossification, meaning that cartilage ossification initially occurs within the perichondrium and then continues progressively from outside to inside. It is important to note that the nascent bone still remains a rod-like shaped cartilage in the centre [49]. 

In the zebrafish lower jaw, the perichondral ossification of Meckel’s cartilage is initiated on the anterior, labial side. The bulkiest bone arising from Meckel’s cartilage via perichondral ossification is the anguloarticular [49]. Nonetheless, most parts of Meckel’s cartilage are encased within intramembranous bone, such as the dentary bone. In the upper jaw, palatoquadrate cartilage turns to bone and in the posterior region gives rise to the endochondral bone, the quadrate, which articulates with the anguloarticular in the jaw hinge region. Conversely, the upper jaw is composed of two dermal bones in the anterior region, premaxilla and maxilla [50]. In contrast with teleosts, there is an evolutionary trend towards the reduction and/or fusion of skeletal elements within mandibular and hyoid arches in mammals. Teleost jaws are composed of a large amount of individual bones which are mostly clearly identifiable. 

Similar to Meckel’s, the ossification of ceratohyal cartilage in the ventral region of the hyoid arch starts within the perichondrium, progressing in the anteroposterior direction. In the dorsal region of the hyoid arch, the hyosymplectic cartilage ossifies into the hyomandibular bone. The hyomandibula is fused with the symplectic bone, which itself derives from the hyosymplectic cartilage in the middle region. Moreover, the hyomandibula articulates with the opercular series, which is composed of several intramembranous bones, including the opercle. Collectively, the intramembranous bones of the opercular series serve as a protection of gill slits. Meanwhile, subsequent branchial arches also ossify perichondrally [50] Opercular intramembranous bones are in sharp contrast with the mammalian hyoid arch, as NCCs in the murine hyoid arch are incompetent at forming intramembranous bone under normal conditions. A summary of skeletal derivates of PAs in the mouse and zebrafish can be found in Table 1. 

In teleosts, the cranial skeleton is highly developed, and the function of pharyngeal cartilage is more akin to the ancestral gnathostome state in comparison with amniotes, as each PA-derived element reflects the adaption to aquatic life [51]. The number of PAs in aquatic species is usually higher, since the gills are relatively inefficient filters. There appears to be a general trend towards the reduction of PAs during evolution. Fossil fish have high numbers of PAs, and there have even been ostracoderm fossils with as many as 30 arches [52]. One reason for the general decrease in PA number in amniotes could be the transition from an aquatic to land dwelling lifestyle. However, it is important to note that the anterior–posterior and dorsal–ventral PA identity and polarity has largely been conserved among all gnathostomes [21]. 

## 3. Specification of Pharyngeal Arches by the *Hox* Code

The anatomical identity of individual PAs is dependent on their position along the anterior-posterior axis. The axial identity of PAs is determined by the expression of *Hox* genes in the hindbrain and in migrating NCCs [53,54]. However, even crestless PAs have a sense of individual identity [15]. *Hox* genes control the segmentation of the hindbrain by the principle of collinearity, meaning that they are organized in clusters in the chromosomes in the same order, as is their expression along the anterior–posterior axis [55]. Cranial NCCs populate PAs in distinct segregated streams, which are defined by the spatiotemporal expression of *Hox* genes in the hindbrain [56]. The neuroepithelium of the hindbrain is transiently subdivided into a series of eight metameric segments, called rhombomeres (r1–r8) [57]. NCCs arising at the level of rhombomeres colonize PAs, which are worth to note also metameric [9]. While NCCs migrating from the level of the forebrain, midbrain, and anterior hindbrain do not express *Hox* genes, those that arise at the level of r3–r8 are *Hox*-positive. According to this anterior–posterior specification, NCCs colonizing the prospective face and the mandibular arch are *Hox*-negative, whereas the hyoid and posterior PAs are *Hox*-positive [58]. In humans and mice, four *Hox* paralogue groups, numbered *Hox1*, *Hox2*, *Hox3*, and *Hox4*, are expressed in the head and neck. Each paralogue group contains *Hox* genes from at least two *Hox* clusters—*Hoxa*, *Hoxb*, *Hoxc*, and *Hoxd*. For example, *Hox1a* and *Hox1b* collectively form one paralogue group, and they both come from two distinct clusters. Due to teleost-specific duplication, as much as seven hox clusters appear in the zebrafish genome—*hoxaa*/*hoxbb*, *hoxba*/*hoxbb*, *hoxca*/*hoxcb*, and *hoxda*/*hoxdb* [59]. Generally, each PA is governed by one *Hox* group—the second PA is controlled by *Hox2*, the third PA by *Hox3*, and the fourth PA by *Hox4* [60,61]. *Hoxa1* itself is not expressed in migrating NCCs but solely in their precursors at the neural plate prior to NCC delamination, and *Hoxa1* lineage gives rise to all NCCs that emanate from r4 [62,63]. Likewise, the expression of *Hoxb1* is apparent only in the neuroepithelium and is very temporary in the mouse [56]. In the zebrafish, *hoxb1* in conjunction with other transcription factors modulates NCC activity in streams migrating from r4 [56]. Mice lacking *Hoxa1* show a significant decrease in migratory NCCs in the second PA and the reduction of the NCC number is even stronger in *Hoxa1* and *Hoxb2* double-null embryos, which lack any NCCs from r4, a major site of origin of the second PA neural crest [14]. In zebrafish, overexpression of *hoxa1* results in robust and partially duplicated ceratohyal cartilages, while the remaining PAs, including the mandibular arch, are underdeveloped [64]. Interestingly, single *Hoxb1*-null mouse embryos display no discernible defects in NCCs [14,65]. 

In contrast, *Hoxa2* has a more direct effect on the craniofacial morphogenesis, since *Hoxa2* is expressed in NCCs emanating to the second, third, and fourth PAs. Strictly speaking, *Hoxa2* is a key determinant of the second PA fate in the mouse [66]. *Hoxa2*-null mice exhibit a homeotic transformation of the first arch derivatives into the second arch skeletal elements [67,68]. Although not studied in the mouse, the ectopic activation of *Hoxa2* in the mandibular arch of fish, frog, and chick transforms the identity of the first PA elements into that of the second arch [69,70,71]. Phenotypic changes in *Hoxa2* mutants suggest that *Hox* genes are incompatible with the mandibular arch development, and this idea is further supported by mutants missing the entire *Hoxa* cluster. In these mouse mutants, the individual identities of the second, third, and fourth PAs are diminished and all are transformed the into rudimentary first PA elements, while posterior PA derivatives do not develop altogether [72]. Nonetheless, this is not firm evidence that the first PA represents a ground state, and the formation of successive PAs requires the *Hoxa* cluster. Akin to *Hoxb1*, mouse *Hoxb2* mutants have only mild craniofacial defects, and their pharyngeal skeletal elements appear normal. In zebrafish, *hoxb2* is expressed only in NCCs emanating from r4 to the second PA, and its individual function is not necessary for hyoid arch development. However, it is important to note that *hoxa2* alone does not drive the development of second PA derivatives in the zebrafish [70]. During the second PA morphogenesis in zebrafish, the combined action of *hoxa2* and *hoxb2* patterns the nascent hyoid arch, as *hoxa2*/*hoxb2* double knockdown changes the morphology of the second PA derivates so that they appear similar to the mandibular arch-derived elements. Altogether, data from *Hox* mutants suggest that the *Hoxa* gene cluster has a primary role in the specification of the axial identity of the PAs, whereas *Hoxb* cluster may serve as a fine tuner of the nascent PA morphology.

## 4. Specification of Mandibular and Hyoid Arches by the MEIS/PBX Complex

MEIS and PBX transcription factors are regulatory proteins containing TALE (three-amino-acid-loop extension) homeodomain. MEIS binds PBX, among other transcription factors, and they collectively form a complex that binds to a DNA via respective MEIS- and PBX-consensus binding sites [73]. Mice and humans possess three *Meis* paralogues—*Meis1*, *Meis2*, and *Meis3*. In zebrafish, *meis1* and *meis2* genes were duplicated during teleost evolution, and its genome contains *meis1a*/*meis1b* and *meis2a*/*meis2b*. When it comes to *Pbx*, mice and humans have four *Pbx* genes *Pbx1*, *Pbx2*, *Pbx3*, and *Pbx4*, whereas the *pbx* family in the zebrafish genes consists of *pbx1a*, *pbx2*, *pbx3*, and *pbx4*, whose function is more akin to murine *Pbx1* [74].

MEIS and PBX transcription factors serve important roles by interaction with HOX proteins during development of the hindbrain and NC. MEIS factors bind to the PBX–HOX complex, therefore forming a stable trimeric complex, allowing the modulation of Hox expression [73,75]. Crosstalk between MEIS and HOX is likely required for the determination of PA identity. In zebrafish, the Meis–Pbx–Hox complex regulates chromatin accessibility in *hoxb1a* and *hoxb2a* gene loci, thereby regulating their expression in the second PA [76]. Correspondingly, the murine MEIS–HOXA2 complex regulates the identity of the second PA by controlling the expression of second PA-specific genes [77]. The elimination of *Meis2* specifically in NCCs results in extensive craniofacial defects [78]. Furthermore, NC-specific *Meis2* embryonic mutants have elevated osteogenesis in the mandibular and hyoid arch at the expense of disrupted tongue development [79]. Craniofacial defects in the maxillary and mandibular processes of *Meis2*-deficient embryos thus reveal the Hox-independent function of Meis2. Furthermore, altered osteogenesis within the hyoid arch also results in various defects of the hyoid apparatus in *Meis2*-deficient embryos [78,79]. The MEIS–PBX–HOX regulatory circuit seems to be evolutionary conserved. In a clinical setting, human patients with heterozygous mutations in *MEIS2* are afflicted by craniofacial and cardiac defects, in addition to intellectual disabilities [80,81,82,83,84,85]. In both lamprey (cyclostomes) and zebrafish (gnathostomes), the deletion of evolutionary conserved *hoxa2* and *hoxb2* enhancers results in loss of *hox* expression in the second migratory stream of NCCs, which contains precursors of second PA cartilage [86]. Intriguingly, the targeted deletion of conserved meis- and pbx-binding sites in these *hox* enhancers leads to the same result. The combined knockdown of *meis1* and *meis2* leads to malformations of craniofacial cartilage, e.g., a fusion of viscerocranial cartilages, demonstrating the importance of *meis* in cranial NCCs [87]. This indicates the improper specification of PAs at the earliest stages, which may affect subsequent steps of cartilage formation. 

Zebrafish Pbx4, the functional equivalent of mammalian PBX1, cooperates with HOX in PA segment setting, as *pbx4* mutants exhibit hypoplastic jaws and the fusion of first and second PA skeletal derivatives [88]. In contrast to zebrafish, murine *Pbx1* is expressed in the ectomesenchyme and ectoderm of the second arch, while maxillary and mandibular processes show much lower expression. Nonetheless, *Pbx*-null and *Pbx2* heterozygous mutants have been reported to exhibit mandibular hypoplasia [89]. Compound *Pbx1*/*Pbx2* mutant mice show abnormal forebrain development, hindbrain segmentation, and hypoplasia of posterior PAs [90]. Mice with systemic elimination of *Pbx1* have morphological alternations of splanchnocranial cartilage derived from the second PA, which mimics the homeotic transformation in *Hoxa2*-null mice. Both lesser horns of the hyoid and styloid processes of the temporal bone develop elongated outgrowths that are fused together. This newly formed cartilaginous structure is oddly reminiscent of the hyoid apparatus of certain nonhuman mammals and of Eagle’s syndrome in humans. Additionally, *Pbx1* mutants lack stapes, another skeletal element derived from the second PA [91]. Elongation of the styloid process or calcification of the stylohyoid ligament above a specific threshold is a medical condition called Eagle’s syndrome. Alongside *Hoxa2* and *Pbx1*-null mutants, calcification or chondrification of the stylohyoid ligament resembling human Eagle’s syndrome can also be observed in *Meis2* and *Prrx2* mutants [79,92]. Calcified stylohyoid ligament conspicuously resembles the hyoid apparatus of some nonhuman mammals, in which it may consist of more parts than in humans. It has been hypothesized that the elongated styloid process in humans is evolutionary coded and represents a form of atavism of the bony hyoid apparatus of our evolutionary ancestors. Clinically, the condition is characterized mostly by pain in the head and neck due to compression of the surrounding structures either by elongation or angulation of enlarged styloid process [93]. Multiple aetiologies of Eagle’s syndrome have been suggested in the literature, ranging from genetic, developmental, endocrine, traumatic, degenerative, and metaplastic. To summarize, the MEIS/PBX complex regulates cell specification within the mandibular arch, whereas the trimeric complex MEIS/PBX/HOX determines cell identity within the hyoid arch. 

## 5. Endothelin–Dlx–Hand Gene Regulatory Network Controlling Anatomical Axes in Mandibular and Hyoid Arches 

### 5.1. Mouse

Already at the onset of PA formation, molecular signals determine the pattern and polarity of the respective arch. The mandibular arch contains *Hox*-free NCCs, so its molecular determination is dependent on distinct signalling cascades, primarily on the Endothelin–Dlx–Hand regulatory network. Endothelin1 (EDN1) is a peptide ligand that binds to G protein-coupled receptor EDNRA and together with its downstream components, DLX and HAND, governs the patterning of jaws [94,95,96]. Both EDN1 and its receptor EDNRA are required for the induction of *Dlx* and *Hand* expression in the mandibular arch [97,98,99]. *Edn1* is expressed in the epithelium, in the paraxial mesoderm, and in the aortic arch vessel endothelium of the mandibular arch, whereas *Endra* is extensively expressed in the ectomesenchyme of the head [100,101]. *Edn1*- and *Ednra*-null mouse embryos exhibit a homeotic transformation of the lower jaw to an upper jaw identity [101,102]. Similarly, ventral structures of the hyoid arch (lesser horns) appear more severely affected in comparison to the dorsal structures (stapes) in *Edn1* mutant mice [103]. Moreover, *Edn1*-null mouse mutants display the absence of the styloid process, and the hyoid bone is largely deformed and fused to the pterygoid process. Conversely, ectopic activation of *Ednra* in the cranial NCCs leads to homeotic transformation of the maxilla into the mandible-like structure [104]. In line with this, misexpression of *Edn1* in the maxilla induces the ectopic dentary bone in the upper jaw region, again demonstrating the reversal of the molecular switch [104]. Cranial NCCs within the mandibular arch are competent at forming both maxilla and the mandible, and *Edn1* is a molecular switch responsible for the choice of the mandibular-specific morphogenetic program [104]. Intriguingly, even indirectly induced ectopic *Edn1* signalling in *Six1*-null mice present in the proximal end of the mandibular arch leads to the formation of rod-shaped bone at the zygomatic arch with a cartilaginous tip [105]. 

A close relationship among *Edn1* and its downstream targets *Dlx* and *Hand* has been proposed in several loss-of-function studies. The combined loss of *Dlx5* and *Dlx6* causes the homeotic transformation of the lower jaw into the maxilla-like structure, which essentially phenocopies *Edn1* knockout [102,104,106]. Furthermore, misexpression of *Hand2* in the *Ednra* domain of the cranial NCCs causes similar transformation to ectopic *Ednra* activation [107]. Altogether, the EDN–DLX–HAND regulatory network is a prime regulator of anterior–posterior (synonymous with ventral–dorsal in the zebrafish) patterning of the mandibular arch and, in that sense, upper and lower jaw identity [104].

**Figure 1:**
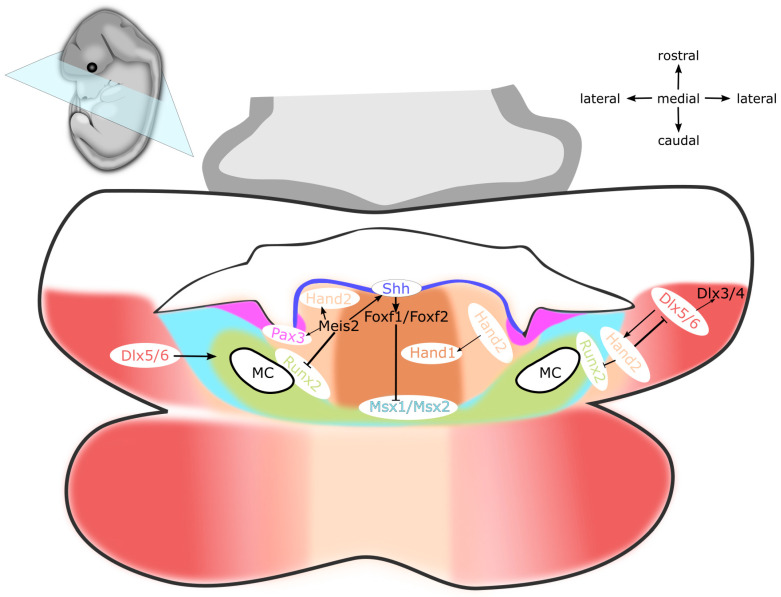
Schematics of a frontal section through developing oral cavity of a mouse embryo at E12. *Dlx5/Dlx6* are expressed in ectomesenchyme of the mandibular process and the hyoid arch. *Hand1/Hand2* and *Meis2* are expressed in ectomesenchyme in the medial region of the mandibular process and the hyoid arch, which also includes the lingual ectomesenchyme. *Msx1* and *Runx2* are expressed within the primordium of prospective dentary bone. *Pax3* is expressed in the ectomesenchyme around the alveolingual sulcus, which represents an anatomical boundary between the dentary bone and tongue. *Shh* is expressed in the lingual epithelium, whereas *Foxf1/Foxf2* are expressed in the lingual ectomesenchyme. Abbreviations: E, embryonic day; MC, Meckel’s cartilage. Expression domains of genes written in black text are not highlighted in the figure.

*Dlx* genes are homeodomain transcription factors that control the intra-arch polarity of pharyngeal arches and anterior–posterior and proximal–distal patterning [106]. In mice, they are organized as three bigene pairs, namely *Dlx1*/*Dlx2*, *Dlx3*/*Dlx4*, and *Dlx5*/*Dlx6*, in the proximity of *Hox* genes in the chromosomes [108,109]. In both mice and zebrafish, there are six *Dlx* genes that are expressed in the ectomesenchyme of the mandibular and hyoid arches. While *Dlx1*/*Dlx2* are expressed almost throughout the entire first two arches, *Dlx3*/*Dlx4* and *Dlx5*/*Dlx6* show more restricted domains [110]. *Dlx5*/*Dlx6* are expressed solely in the mandibular process and hyoid arch (see Figure 1), in the nested domains within *Dlx1*/*Dlx2* territory, whereas *Dlx3*/*Dlx4* are expressed only in the most distal part of the mandibular process and hyoid arch, within *Dlx5*/*Dlx6* territory. 

Single and compound *Dlx1*/*Dlx2* mouse mutants display malformations selectively in the upper jaw and upper hinge region, with barely any effect in the lower jaw [110,111,112]. Many anterior first PA elements, such as alisphenoid and incus, are malformed in the *Dlx2*-null mice, whereas *Dlx1*-null mutants exhibit a similar phenotype, although much milder. However, compound double-null *Dlx1*/*Dlx2* mutants develop more severe defects that are not present in either of the single-null mutants [111,112], suggesting their functional redundancy for the development of maxilla. Furthermore, *Dlx1*/*Dlx2* may be dispensable for the development of the lower jaw, as there are barely any malformations in the lower jaw associated with *Dlx1*/*Dlx2* single or compound mutations. Single *Dlx2* and compound *Dlx1/Dlx2* mouse mutants exhibit no abnormalities in the ventral region of the hyoid arch, although they display cleft hyoid bodies and fusion of the greater horns to the superior horns of the thyroid cartilage [110]. Interestingly, although the expression of *Dlx2* is unaltered in the mandibular process of *Edn1* mutants, it is slightly diminished in the hyoid arch [103]. On the other hand, mice with targeted deletion of *Dlx5* have lower jaw defects, particularly hypotrophy and dysmorphy of Meckel’s cartilage [113,114]. In double-null *Dlx5*/*Dlx6* mice, the shape of maxillary and mandibular processes is identical during embryogenesis, and the lower jaw never develops Meckel‘s cartilage, but mouse whiskers arise on its surface [110,115,116]. Concomitantly, compound *Dlx5/Dlx6* mouse mutants exhibit the truncated styloid process with an ectopic process extending towards it from the hyoid bone and lesser horns projecting towards the neurocranial base [110]. The forced expression of *Dlx5* in NCCs in the maxillary process leads to upregulation of mandibular-specific genes and appearance of several phenotypic hallmarks of the mandible in the maxilla region [106]. This represents the aforementioned homeotic transformation of the lower jaw into the upper jaw-like structure, which suggests that the default state of the jaw is maxillary, and EDN–DLX–HAND is required to initiate the lower jaw development programme. The specific function of *Dlx3*/*Dlx4* during the development of the mandibular arch remains elusive, since no craniofacial phenotype has been described in *Dlx3*-null mice, and *Dlx4*-null mice have not been reported yet [117]. However, *Dlx3*/*Dlx4* is induced by *Dlx5*/*Dlx6* [116,118], and their functional redundancy cannot be excluded. In summary, combinatorial *Dlx* expression domains within PAs make up a prerequisite for intra-arch identity of individual skeletal elements along the proximal–distal axis [37,110]. 

Hand basic helix-loop-helix transcription factors are expressed in the distal region of the mandibular process, where they act to specify the so-called distal tip. *Hand2* is regulated by *Dlx5*/*Dlx6*, which are induced by EDN1 signalling (END–DLX–HAND), therefore specifying the mandibular identity (see Figure 1). The view that *Hand2* expression is not compatible with maxillary development is further supported by Sato et al., who show that ectopic *Hand2* expression transforms the maxilla into the mandible [104]. Of note, *Dlx5*/*Dlx6* and *Hand2* are severely reduced in the hypoplastic mandibular process of *Mef2c* NC-specific mutants, which links *Mef2c* to the Edn–Dlx–Hand regulatory network [119]. Contrary to *Edn1* induced by the expression of *Hand2*, the expression of *Hand1* requires BMP signalling. Moreover, HAND2 acts synergistically with BMP to regulate the expression of *Hand1* [120,121], since *Hand1* expression is markedly downregulated in *Hand2* mutants. *Hand1* and *Hand2* are expressed in the distal tip domain of the mandibular process and hyoid arch, which is mutually exclusive with the more proximal expression domain of *Dlx5*/*Dlx6.* Tissue-specific inactivation of *Hand2* in NCCs leads to ectopic ossification in the distal tip of the mandible, heterotopic bone in the symphysis, and tongue hypoplasia [120,122]. Similarly, the deletion of the branchial enhancer of *Hand2* in the mandibular arch leads to the hypoplasia of the mandible and cartilage malformations, such as truncation of Meckel’s cartilage and abnormal projections of the malleus and lesser horns of the hyoid [107]. Multiple defects of the hyoid apparatus have been reported to occur in *Hand* mutants, including poor ossification of the hyoid bone and lesser horns, deformation of the hyoid body in the midline, fusion of the hyoid body and thyroid cartilage in the midline, fusion of lesser horns and palatine bones, and aberrant articulation of the styloid process with greater horns [120,123,124,125]. *Meis2* appears to act upstream of *Hand2* because NC-specific *Meis2* mutants exhibit decreased *Hand2* expression in the first and second PAs. In *Wnt1*-*Cre2*-driven, tissue-specific deletion of either *Meis2* or *Hand2* in NCCs, mutant mice show comparable tongue hypoplasia, mandibular retrognathia, and symphyseal ossification [79,120]. The expression of *Hand* genes in the distal tip of the mandibular process thus restricts osteogenesis in the prospective tongue region, while *Dlx* genes ensure the development of individual pharyngeal skeletal elements in the proximal region [121]. Altogether, BMP simultaneously with EDN1 acts to divide the nascent mandibular process into the tongue-forming *Hand*-positive nested domain and the bone-forming *Dlx*-positive nested domain. 

### 5.2. Zebrafish

**Figure 2:**
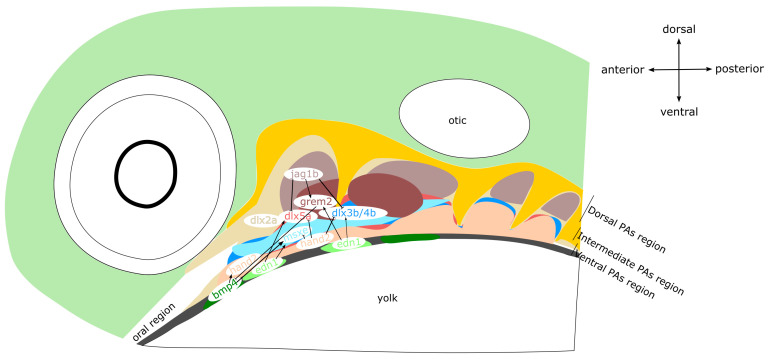
Schematic model of PA patterning along the dorsal–ventral axis in zebrafish at 30–36 hpf (hours post-fertilization). Lateral view, head to the left, pharyngeal endoderm in dark yellow, pharyngeal ectoderm in dark grey. The dorsal region of PAs is established by co-operative action of *jag1b* and *grem2* and characterized by the expression of *dlx2a*. The intermediate region of PAs is controlled by *dlx3b/4b/5a* and *msxe,* which are downregulated by *edn1* in the pharyngeal ectoderm. The ventral region of PAs is dominated by the expression of *bmp4-hand2*.

In the zebrafish, *edn1* is expressed in the pharyngeal ectoderm, mesoderm, and endoderm. However, only ectodermal *edn1* seems to control the fate of NCCs during the formation of the intermediate-ventral region of PAs (see Figure 2). Thus, mutations *in edn1* lead to hypoplasia of the Meckel’s cartilage and its fusion with the palatoquadrate cartilage [126]. In addition, both *edn1* mutants and *edn1* morphants have malformed intramembranous bones within the mandibular and hyoid arches [127]. The mutant phenotype is also reflected in the alteration of the molecular imprint, as *edn1* mutants have decreased expression of *hand2*, *dlx2a*, *msxE*, and *gsc*, especially in the ventral region of PAs [128], while *nkx3.2* in the jaw hinge region is also reduced [129]. The essential role of *edn1* during patterning of the ventral region of PAs was confirmed by heat-shock experiments, resulting in disrupted expression of *edn1,* the reduction of *hand2*, and the simultaneous expansion of *dlx3b*, *dlx5a*, *dlx6a*, and *nkx3.2* [130]. Unlike in the mouse, *edn1* does not recognize one but two paralogue receptors, *Ednra1* and *Ednra2*. *Ednra1* is expressed in the migratory and early postmigratory NCCs within PAs, whereas *endra2* is expressed in the late postmigratory NCCs [126]. *Ednra1* knockdowns display the fusion of joints in hinge regions of the mandibular and hyoid arches, as well as retrognathia. Unlike *ednra1*, knockdown of *ednra2* does not affect PA development. Moreover, *ednra1/2* double knockdown mutants miss the lower jaw and ceratohyal cartilage, similar to *edn1* mutants [126]. Thus, Edn1 signalling via *Ednra1* and *Ednra2* is important during development of the ventral region of PAs. [131]. Collectively, data from mice and zebrafish suggest the evolutionarily conserved function of *edn1* in postmigratory NCCs and during the development of ventral pharyngeal cartilages in gnathostomes. 

The craniofacial phenotypes of *edn1* and *hand2* mutants appear to be similar. *Hand2* zebrafish mutants lack the lower jaw and ventral set of second pharyngeal cartilages [129]. In fact, *edn1* positively regulates *hand2* during the development of ventral pharyngeal cartilages. Upon the early NC migration, *hand2* restricts cell proliferation during the anterior-ventral protrusion of NCCs, which is under the control of *edn1*. However, at later stages of development, the function of *hand2* shifts, and it eventually promotes the cell proliferation [131]. Additionally, *hand2* also influences the cell movement within the mandibular arch, but apparently independently of *edn1* [131]. *Nkx3.2* is expressed in ectomesenchyme of the lower jaw primordium, and during the chondrification, its expression becomes localized within and around the jaw joint. In keeping with this, *hand2* regulates development of the jaw joint via modulation of *nkx3.2* expression [129,132]. Therefore, n*kx3.2* is involved in specification of the intermediate region of PAs, the hinge region, and is expressed ventrally to *dlx2a* and dorsally to *hand2* [129]. The expression of *nkx3.2* in the presumptive jaw hinge region is regulated by Hand2 via *gsc* and *dlx3b/4b/5a.* Hand2 activates the expression of *gsc*, which in turn represses *nkx3.2*. Meanwhile, *dlx3b/4b/5a* repress *gsc* and activate *nkx3.2* [132].

Dlx genes are under the control of Edn1 and Bmp signalling [133]. The genome of zebrafish contains four bigene dlx pairs—*dlx1a*/*dlx2a*, *dlx3b*/*dlx4b*, *dlx5a*/*dlx6a*, and *dlx2b*/*dlx4a* [134]. Along the dorsoventral axis of PAs, *dlx3b*/*dlx4b* and *dlx4a* can be detected in the intermediate region. While *dlx2a* is expressed in the dorsal region of PAs, *dlx5a*/*dlx6a* are found in the ventral region and the intermediate region of PAs (see Figure 2). The expression of *dlx2b* is excluded from the first two PAs. In the ventral region of PAs, *dlx* genes are repressed by *hand2* [132]. Double knockdown of *dlx1a*/*dlx2a* causes defects in the dorsal pharyngeal cartilages (palatoquadrate and hyosymplectic cartilages), bearing similarities to murine *Dlx1*/*Dlx2* mutants [110,111,112]. The patterning of mandibular and hyoid arch hinge regions, the opercle, and branchiostegal rays is influenced by *dlx5a*, *dlx3b*, and *dlx4b*. Interestingly, single knockdown of *dlx5a*, *dlx3b*, or *dlx4b* does not produce any changes in the pattern of expression. Conversely, simultaneously knocking down all of them leads to the loss of hinge region joints and fusion of the opercle with branchiostegal rays [132]. Taken together, *dlx1a* and *dlx2a* control the patterning of the dorsal region in the mandibular and hyoid arches, whereas both *dlx3b*/*dlx4b* and *dlx5a*/*dlx6a* regulate the development of intermediate region. The expression of *dlx* genes in the zebrafish is in accordance with the mouse, as *Dlx1/Dlx2* govern morphogenesis in the dorsal region of PAs, which equals the upper jaw region, the styloid process, and the stapes, whilst *Dlx3/Dlx4* and *Dlx5/Dlx6* establish the intermediate and ventral region of PAs, which comprises the presumptive lower jaw and lesser horns of the hyoid. Therefore, the patterning along the anterior–posterior and dorsal–ventral axes in mice and zebrafish is under the control of a common regulatory cascade, EDN–HAND–DLX (see Figure 1 and Figure 2). 

## 6. Combinatorial Action of FGF8, BMP4, and SHH Signalling Pathways during Morphogenesis of Mandibular and Hyoid Arches 

### 6.1. Mouse

**Figure 3:**
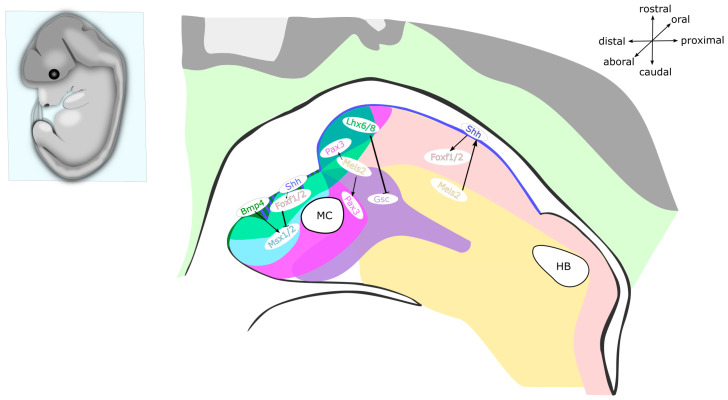
Schematics of a mid-sagittal section through the mandibular and hyoid arches of a mouse embryo at E12. *Bmp4* is expressed in the distal mandibular epithelium, at the site of presumptive incisors, while *Msx1* is expressed in the distal mandibular ectomesenchyme, surrounding the incisor primordia. *Shh* is expressed in the vestibular lamina and dental epithelium, as well as in the lingual epithelium, while *Foxf1/Foxf2* are expressed in ectomesenchyme in the medial region of the mandibular process, surrounding the incisor primordium and inside the nascent tongue. *Pax3* is expressed in the ectomesenchyme of distal tip of the mandibular process and nascent tongue. *Lhx6/Lhx8* are expressed in ectomesenchyme on the rostral side, while *Gsc* is expressed on the caudal side of the mandibular process. *Meis2* is expressed in the medial proximal region of the mandibular process and in the medial region of the hyoid arch. Abbreviations: E, embryonic day; MC, Meckel’s cartilage; HB, cartilage primordium of the hyoid bone. Expression domains of genes written in black text are not highlighted in the figure.

**Figure 4:**
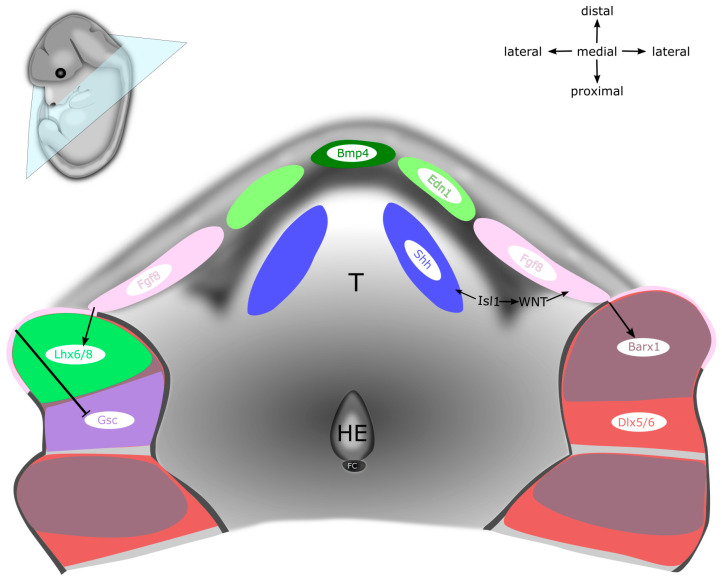
Schematics of the mandibular and hyoid regions of a mouse embryo at E12, superior view at the nascent tongue from inside of the alimentary canal. *Bmp4* and *Edn1* are expressed in the distal mandibular epithelium, while *Bmp4* also marks the site of presumptive incisors. *Shh* is expressed in lingual epithelium of the tongue primordium. *Fgf8* is expressed in the proximal mandibular epithelium, at the site of presumptive molars, while *Barx1* is expressed in the proximal mandibular ectomesenchyme, surrounding the molar primordia, and in ectomesenchyme of the hyoid arch. *Dlx5/Dlx6* are expressed in ectomesenchyme of the mandibular process and the hyoid arch. *Lhx6/Lhx8* are expressed in the ectomesenchyme on the rostral side, while *Gsc* is expressed on the caudal side of the mandibular process. Abbreviations: E, embryonic day; T, tongue primordium; HE, hypobranchial eminence (second PA); FC, foramen caecum. Expression domains of genes written in black text are not highlighted in the figure.

Alongside transcription factors, numerous protein ligands also serve essential functions during the patterning of mandibular and hyoid arches. *Ffg8* is expressed in the oral epithelium from which it diffuses into the underlying ectomesenchyme. *Fgf8* is a key survival factor of the NC because its ablation in the oral ectoderm leads to massive apoptosis in the mandibular arch, as well as to complete loss of the proximal mandibular structures [135]. Moreover, *Fgf8* determines the rostral–caudal axis of the mandibular arch. The expression of *Fgf8* in the oral surface ectoderm induces the expression of transcription factors *Lhx6*/*Lhx8* in the rostral mandibular mesenchyme. Concomitantly, this results in the restriction of Goosecoid (*Gsc*) expression in the caudal mandibular mesenchyme, therefore establishing the subdivision of the mandibular process into the rostral and caudal domain (see Figure 3 and Figure 4) [136]. At the same time, FGF8 acts together with BMP4 to specify the proximal–distal axis by regulating the expression of specific homeodomain-containing transcription factors in the ectomesenchyme, which subsequently defines the positional identity of individual teeth. *Barx1* induced by FGF8 in the proximal region determines the molar identity, whereas BMP4-regulated *Msx1* in the distal aboral region specifies the prospective incisors. Intriguingly, early mandibular epithelium can organize dental mesenchyme and dental papilla in the mouse hyoid arch, indicating a common regulatory circuit between the mandibular and hyoid arches during the early stages of PA development [137]. In the mandibular process, maintenance of *Fgf8* expression is ensured by transcription factor PITX2, which simultaneously represses *Bmp4* expression. Consistently, the expression of *Fgf8* and its target genes, such as *Barx1* and *Pitx1*, is severely reduced in *Pitx2*-null mutants, whereas the expression of *Gsc* in the mandibular process is expanded rostrally. Moreover, since high doses of *Pitx2* are required for repression of BMP signalling, the expression of *Bmp4*, *Msx1*, and *Msx2* is expanded as well. As a result, disrupted signalling in the mandibular arch due to the mutation in either *Pitx1* or *Pitx2* leads to a severe micrognathia, while single *Pitx1* mutants also suffer from the bifurcation of the tongue and a novel bone deposition around Meckel’s cartilage [138,139,140]. 

The rostral–caudal axis, which is defined by the complementary expression of *Lhx6*/*Lhx8* and *Gsc,* is actually distinct from the oral–aboral axis (see Figure 3 and Figure 4) [141]. Corresponding to the expression pattern of Sonic hedgehog (*Shh*) in the oropharyngeal epithelium, the downstream targets and mediators of Hedgehog (HH) signalling, *Foxf1* and *Foxf2* are expressed in the subjacent mandibular mesenchyme [141,142]. Complementary to the expression of *Shh* on the oral side of the mandibular arch, *Bmp4* is expressed in the complementary subdomain on the aboral side (the same domain that is important for the development of incisors) [141,143]. The expression of *Foxf1*/*Foxf2* genes in the mandibular mesenchyme antagonizes the expression of *Msx1*/*Msx2* induced by BMP4, thereby preventing the osteogenesis in the prospective tongue region. Upon ablation of either *Smo* or *Foxf1*/*Foxf2* in NCCs via *Wnt1-Cre2* recombination, the *Bmp4* expression domain expands to the oral side of the mandibular arch, which leads to the formation of heterotopic bone on the oral side of the mandible [141]. Altogether, this shows that HH signalling in the mandibular arch is required for patterning the oral–aboral axis of the mandible. 

Transcription factor MEIS2 modulates SHH activity in the mandibular process and determines its medial–lateral axis [79]. The targeted deletion of *Meis2* in the NC using *Wnt1*-*Cre2* driver leads to the downregulation of *Shh* and *Ptc1* expression on the oral side of the mandibular process. Furthermore, the expression of *Hand1*/*Hand2* in the distal tip of the medial region of mandibular and hyoid arches is reduced, while the gradient of *Dlx5* and *Barx1* expands from the lateral to medial regions. This patterning shift along the medial–lateral axis leads to the loss of molecular identity of NCCs in the prospective tongue. When Meis2 is deleted within NCCs in Wnt1-Cre2; *Meis2* ^fl/fl^ mutants, the levels of PAX3 around the alveolingual sulcus (anatomical boundary between dentary bone and tongue) are markedly reduced and replaced with RUNX2, which subsequently leads to the formation of ectopic bone in the same region. As a result, the tongue is severely hypoplastic and its lateral edges are invaded by heterotopic bone. Altogether, the determination of the oral–aboral and medial–lateral axes in the mandibular process by the coordinated interaction of SHH, BMP, and the EDN–DLX–HAND regulatory cascade may be linked to the MEIS2 regulatory network, since its ablation in the NCCs leads to the downregulation of both *Shh* and *Hand2*. 

Shortly after the colonization of PAs by NCCs, *Shh* is expressed in the oropharyngeal epithelium, from which it maintains the survival, proliferation, and patterning of the underlying mandibular mesenchyme. Both epithelial and mesenchymal cells in the mandibular arch express receptors *Smo* and *Ptch1* and are therefore able to respond to SHH ligand. As development of the mandibular arch proceeds, spatially restricted centres of *Shh* induce the formation of numerous oral structures, including tongue, teeth, palate, and salivary glands (see Figure 1, Figure 3 and Figure 4) [144,145]. The elimination of SHH activity in either oropharyngeal epithelium via *Nkx2.1-Cre,Shh^flox^* or SHH responsiveness in the ectomesenchyme using *Wnt1-Cre2;Smo^flox^* causes extensive apoptosis of NCCs and results in mandible and tongue defects [141,142,146,147,148]. At the midline of the mandibular process, the expression of *Shh* specifies NCCs in the tongue primordium, thereby establishing the oral–aboral and medial–lateral axes. Moreover, *Shh* in this region allows the invading myogenic progenitors to permeate the nascent tongue primordium, thereby promoting the tongue development and preventing osteogenic differentiation in the midline [79,141]. Thus far, *Shh* has not been reported to exert any patterning activity in the second PA [149,150]. 

In the early pharyngula, signalling centres expressing *Fgf8* and *Shh* are set up by Islet1 (ISL1). *Isl1* is a member of the *Lhx* family that encodes transcription factors containing two LIM domains and a homeodomain. In the PA development, ISL1 acts as an epithelial ligand expressed in the oral ectoderm of the first PA and the endoderm of other arches [151]. Loss of *Isl1* in β-catenin expressing cells leads to agnathia, a complete absence of the lower jaw [151]. When *Isl1* is inactivated in the mandibular epithelium, specifically in *Shh*-expressing cells, the aberrant bony fusion of the distal tip of the dentary bone occurs, similar to *Hand2* and *Meis2* mouse mutants [79,123]. Both *Fgf8* and *Shh* are missing in the oropharyngeal epithelium of the early pharyngula in *Isl1* mutants [151,152]. Canonical WNT signalling is known to be upstream of *Fgf8* in the first PA epithelium, and WNT signalling is disrupted in the first PA of *Isl1* mutants, indicating a regulatory circuit of Isl1-Wnt-Fgf8 [151,153]. ISL1 may activate epithelial β-catenin signalling via repression of WNT antagonist. Intriguingly, reactivation of β-catenin in the mandibular epithelium of *Isl1* mutants rescued the mandibular morphogenesis through SHH signalling to the mandibular ectomesenchyme. Furthermore, overexpression of *Shh* in the first PA epithelium partially restored the morphologic defect in *Isl1* mutants and led to successful outgrowth of the dentary bone [154]. 

### 6.2. Zebrafish

In the zebrafish, *fgf8* in concert with *fgf3* establishes the segmentation of the pharyngeal endoderm within PAs during the early pharyngula stage [155]. Together, *fgf8* and *fgf3* control the survival of NCCs during the formation of pharyngeal cartilages. Therefore, loss of *fgf8* leads to hypoplasia of the mandibular cartilage [2,155]. Furthermore, *fgf3* knockdown results in misshapen ceratohyal and lack of ceratobranchial cartilages. The complementary function of *fgf8* and *fgf3* is strongly supported by severe pharyngeal malformations in compound *fgf8* and *fgf3* knockdown mutants, which results in loss of all ceratobranchial and hyoid arch cartilages, accompanied by significant size reduction of mandibular arch cartilages [155]. Later in development, *fgf8* is essential for the proper expression of the osteogenic genes *runx2* and *sp7* during the craniofacial ossification [2]. Together with Bmp, Fgf signalling controls the expression of *barx1*. *Barx1* is expressed in migratory NCCs and also later in the ectomesenchyme within PAs, where it maintains the chondrogenic cell fate and negatively regulates the development of the jaw joint, and its loss initiates osteogenic differentiation within chondrocytes [156].

In conjunction with Edn1, Bmp signalling patterns the dorsal–ventral axis of PAs (see Figure 2) [130,133]. Lack of Bmp signalling in PAs leads to either reduction or even loss of ventral pharyngeal cartilages, such as Meckel’s cartilage and ceratohyal cartilage, and intermediate pharyngeal cartilages, such as joints, interhyal cartilage, and the ventral part of symplectic cartilage [133]. Conversely, *bmp* overexpression transforms and fuses hyosymplectic cartilage into a structure reminiscent of ceratohyal cartilage, and joints within mandibular, hyoid arches, as well as the ventral part of hyosymplectic cartilage, are lost. Moreover, palatoquadrate cartilage is also transformed into a structure resembling Meckel’s cartilage [130]. Akin to the loss of Bmp signalling, *edn1* overexpression results in similar defects in dorsal pharyngeal cartilages of the mandibular and hyoid arches, except for the joints. 

In the early pharyngula, the inhibition of Bmp signalling causes the downregulation of Edn1 signalling, as well as downregulation of *hand2* and *dlx6a* in ventral pharyngeal cartilages [133]. At first, Bmp induces Edn1 signalling and restricts the expression of *jag1b* in the dorsal region of nascent PAs. In addition, the joint action of Bmp and Edn1 activates *hand2* via *dlx5a*/*dlx6a* in the ventral region of PAs. After the NC migration, Bmp controls the ventral fate of PAs in an independent manner, whereas *Edn1* regulates the intermediate region of PAs. During this stage, *hand2* in the ventral region is under the exclusive control of Bmp. Furthermore, Hand2 represses intermediate-region-specific genes, such as *nkx3.2*, as well as ventral-region-specific *dlx3b*/*dlx4b*/*dlx5a* [130,133]. The restriction of *bmp* in the ventral region of PAs is mediated by the dorsal-intermedial expression of *grem2*, an antagonist of Bmp signalling, induced by *edn1* and *jag1b* (see Figure 2). In the ventral region of PAs, Bmp inhibits *grem2* expression [130], whereas the intermediate region of PAs is established by the collective action of *dlx3b*, *msxe*, and *nkx3.2* [133]. While *msxe* expression is coregulated by both Bmp and Edn1, *dlx3b* expression is driven solely by Edn1. To sum up, the *grem2*-mediated repression of *bmp* restricts *hand2* to the ventral region of PAs, where Hand2 acts to inhibit the expression of *dlx3b*, *dlx5a*, *dlx6a*, and *nkx3.2* (see Figure 2) [130,133]. Thus, during pharyngeal chondrogenesis, Bmp signalling governs the specification of ventral cartilages, whereas Edn1 regulates the development of intermediate cartilages [130,133]. 

In contrast to zebrafish, FGF, BMP, and SHH set up the position of the prospective tongue and teeth within the murine oral cavity. In zebrafish, these molecules do not play a complementary role, as teeth in zebrafish grow inside the pharynx, not within the oral cavity, and they are not heterogenous, meaning they do not have incisor/molar identity. Moreover, a tongue-like structure in zebrafish is not homologous with the muscular tongue of tetrapods, so SHH signalling at the midline of murine embryos is not readily comparable to that in the zebrafish [157]. However, in both mice and zebrafish, Bmp signalling controls the development of the ventral pharyngeal region, as loss of *bmp* in the zebrafish leads to the lack of ventral pharyngeal cartilages, and ectopic expression of *Bmp* in the mouse results in the duplication of the dentary bone. 

## 7. Molecular Regulation of Osteochondrogenesis in the Mandibular and Hyoid Arches 

### 7.1. Mouse

During the morphogenesis of PA-derived skeletal elements, the differentiation of osteoblasts and chondroblasts from a common osteochondral progenitor represents a critical step towards the formation of bone and cartilage. In regions of prospective cartilage and bone, these osteochondral progenitors aggregate and condense. Both intramembranous and endochondral ossification start from mesenchymal condensations, but the processes themselves are different: during intramembranous ossification, mesenchymal progenitors can differentiate exclusively into osteoblasts, whereas endochondral ossification encompasses the differentiation of both osteoblasts and chondroblasts. The key difference is that chondroblast differentiation precedes the formation of endochondral bone. Osteochondroblastic differentiation and maturation are regulated by three master transcription factors, SOX9, RUNX2, and SP7 (also known as Osterix, OSX). Osteochondral progenitors in early mesenchymal condensation have dual differentiation potential, as they coexpress *Sox9* and *Runx2* [158,159,160,161]. 

During skeletogenic differentiation, WNT signalling is a key regulator of chondroblast versus osteoblast cell fate choice in NCCs. The tissue-specific conditional deletion of β-catenin (*Ctnnb1*), the effector of canonical WNT signalling, results in the complete agenesis of cranial bones [162]. Concomitantly with WNT signalling the inactivation and agenesis of cranial bone, osteogenic progenitors are diverted into the chondrogenic fate, and an ectopic cartilage forms [163,164]. An alternative hypothesis is that RUNX2 and SP7 are intrinsic factors which are not only required for the determination of osteoblastic cell type, but they also play a role in suppressing the differentiation program that leads to chondroblastic cell fate. Cell fate at early stages of differentiation is seemingly still flexible because *Runx2*-expressing osteoblasts still maintain some cell fate plasticity. Full differentiation along the osteoblast lineage is likely ensured by *Sp7*, since in mouse mutants with inactivated *Sp7*, ectopic chondrocytes form at the expense of osteoblasts in some areas where intramembranous bone should form [165]. 

Transcription factor SOX9 is a master regulator of chondrogenesis, and its expression in NCCs is necessary for the formation of craniofacial cartilage. SOX9 probably regulates chondrogenesis by upregulating the expression of *Col2a1* and *Col11a2*, types of collagen found predominantly in the cartilage [158,166]. The tissue-specific deletion of *Sox9* in the NCCs results in loss of all cartilage elements derived from the cranial neural crest. Intriguingly, although the dentary is smaller in *Sox9*-deficient mice, the gross morphology and bone formation are not severely affected [167]. Furthermore, inactivation of *Sox9* in cranial NCCs also results in upregulation of osteoblast marker genes such as *Runx2*, *Sp7*, and *Col1a1* [167]. This further supports the notion that the osteoblastic differentiation programme plays a role in suppressing chondroblastic cell fate, and vice versa. When it comes to the hyoid arch, the specifier of second arch fate HOXA2 regulates the expression of *Sox9*. Under normal circumstances, HOXA2 prevents chondrogenesis in the second PA by suppressing the expression of *Sox9* [66]. When HOXA2 is absent, chondrogenesis is activated ectopically and a duplicated set of first PA cartilages appear in the *Hoxa2* expression domain of the hyoid arch. Thus, the expression of Sox9 in NCCs is required for the differentiation of common osteochondrogenic progenitors into chondroblasts and for the formation of all craniofacial cartilages.

RUNX2 is a transcription factor that controls the differentiation of mesenchymal progenitors (preosteoblasts) into osteoblasts and is expressed in early osteoblasts, hypertrophic Meckel’s cartilage, and mineralized bone [168]. RUNX2 is also a positive regulator of hypertrophic differentiation, as *Runx2*-null mice lack hypertrophic cartilage whatsoever. Systemic deletion of *Runx2* in mice shows that it is important for both intramembranous and endochondral ossification [169]. Loss of *Runx2* in mice leads to total agenesis of bone and a complete loss of expression of osteocalcin and osteopontin, two major non-collagenous proteins in the bone matrix [169]. In the absence of *Runx2* solely in the neural crest, loss of frontal, zygomatic, squamous temporal bone occurs, whereas the dentary, maxilla, premaxilla, and nasal bones are severely hypoplastic and hypomineralized [170]. However, deficiency of *Runx2* in mice not only affects bone but also both the primary and secondary cartilage, as mutant mice lack the condylar cartilage and have deformed Meckel’s cartilage [171]. *Runx2* is controlled by DLX5 and both are essential in driving the differentiation of mesenchymal precursors into osteoblasts. In the prospective tongue region in the mandibular arch, *Hand2* plays a major role in establishing a negative feedback loop in the DLX5/DLX6-RUNX2 circuit. Furthermore, ossification defects in *Runx2*-deficient mice reach beyond the mandibular arch, as the mineralization of the hyoid body is impaired as well. The transition of preosteoblasts into mature osteoblasts is regulated by SP7, a major downstream target of RUNX2. All osteoblasts and even hypertrophic chondrocytes express *Sp7*. Although deficiency of *Sp7* in mice leads to the loss of dentary bone, the development of Meckel’s cartilage is seemingly not affected at all [165]. Interestingly, when *Sp7* is lost exclusively in the neural crest, the dentary bone forms but ends up tiny and rudimentary [172]. To summarize, *Runx2* expression within PAs gives NCCs the potency to form bone, while *Sp7* is required for full commitment to osteoblastic lineage. 

Muscle segment homeobox transcription factors (*Msx1* and *Msx2*) are initially expressed together with *Sox9* in the migrating cranial NCCs. Upon complete colonization of PAs, expression domains of *Msx* and *Sox9* become separate [173]. Until cranial NCC migration within the mandibular process is completed, MSX2 inhibits chondrogenic differentiation of *Sox9*-positive NCCs. In mice and humans, both single *Msx2* and compound *Msx1* and *Msx2* mutations lead to cleidocranial dysplasia with enlarged parietal foramina [174]. This rare genetic condition is characterized by disrupted osteoblast differentiation that clinically presents with hypoplasia of jaw and tooth abnormalities, among many other symptoms. Loss of *Tbx1* specifically in murine NCCs induces a similar phenotype to cleidocranial dysplasia and results in a lack of the hyoid body and fusion to the thyroid cartilage [175]. Generally, the genetic cause of classical cleidocranial dysplasia in humans is heterozygous loss of *RUNX2*, not *MSX2*. However, the hyoid phenotypes of *Runx2^+/−^* and *Tbx1^−/−^* are different, indicating that *Tbx1* might have a primary role in early patterning and perichondral ossification in the hyoid bone [175]. As mentioned before, hyoid anomalies occur in human patients with 22q11.2 deletion syndrome, the clinical picture of which is thought to be caused by loss of the TBX1 gene. Alongside the cleidocranial dysplasia, a mutation of the MSX2 gene in humans can cause craniosynostosis and enlarged parietal foramina, whereas haploinsufficiency can lead to midline cranial defects [176,177,178]. On the other hand, mutations in *MSX1* are connected predominantly with dental abnormalities in humans, such as Witkop syndrome and tooth agenesis [179]. Mutations of *Msx* genes in mice also encompass a wide variety of first and second arch malformations. Overexpression of either wild-type or mutant human *MSX2* in transgenic mice causes mandibular hypoplasia, cleft palate, and decreased ossification of the hyoid, etc. [180]. Single *Msx1*-null mouse mutants display an anomalous malleus, loss of alveolar dentary bone and maxilla, and failure of tooth development. [181]. The combined loss of *Msx1* and *Msx2* in mice results in severe defects such as cleft palate, truncated mandibular process, and decreased volume of trigeminal ganglia [182]. To summarize, during early craniofacial development, *Msx* genes influence the suppression of chondrogenesis and later control the skeletogenic differentiation, as overexpression, misexpression, or deficiency of Msx impedes the osteoblastic differentiation and results in craniofacial bone, cartilage, and tooth defects. 

PAX3 is a transcription factor that is robustly expressed in cranial NCCs that make up the entire palatal, lingual, and mandibular mesenchyme, where it possibly keeps mesenchymal NCCs in an undifferentiated state [183]. Later in development, the mesenchymal expression localizes to the distal tip of tongue and the mandible [79,183]. *Pax3* mutants with persistent *Pax3* overexpression in the entire mandibular arch, including the tongue, display defects in osteogenesis. In NCCs, PAX3 directly regulates the expression of a soluble inhibitor *Sotdc1*, which diminishes responsiveness to BMP and decreases the expression of *Runx2* [183]. In *Meis2* NC-specific conditional mutants, the expression domain of *Pax3* in the tongue is dramatically reduced, whereas the *Runx2* expression domain is expanded in the medial mandibular region, which leads to heterotopic ossification in the lingual mesenchyme [79]. In *Pax3*-deficient Splotch mice, the hyoid bone is often split and partially fused to the thyroid cartilage [184]. In NCCs, *Pax3* seems to be colocalized with *Goosecoid* (*Gsc*) in the postotic NC, frontonasal prominence, mandibular arch, and hyomandibular cleft [185]. *Gsc* encodes a highly conserved homeodomain transcription factor. During cranial morphogenesis, *Gsc* is initially expressed in the undifferentiated tissue of first and second PAs. During PA formation in mouse, *Gsc* expression persists in the nascent lower jaw and tongue, as well as in the hyomandibular cleft [186]. Among many skeletal malformations, *Gsc*-null mice exhibit malformations of malleus, palatine, maxillary, alisphenoid, pterygoid, coronoid, and angular processes [187,188]. Since *Gsc* is also expressed in the hyomandibular cleft, its inactivation results in auditory canal atresia and loss of tympanic rings. In humans, heterozygous loss of *GSC* results in SAMS syndrome (short stature, auditory canal atresia, mandibular hypoplasia, and skeletal abnormalities), which further confirms the role of *Gsc* in craniofacial and joint development. *Gsc* is possibly a downstream effector gene of regulatory networks that defines the specification and cell fate of neural crest and mesodermal lineages. Therefore, dysregulation of GSC-mediated gene expression in the connective tissue results in pathological differentiation and adaptation of new cartilaginous or osseous fate [185]. 

Skeletogenesis in the medial region of the mandibular process is regulated by *Prrx* transcription factors. *Prrx1* and *Prrx2* are expressed in overlapping domains throughout the ectomesenchyme of PAs, with the strongest expression in the mandibular and hyoid arches [189,190]. Of note, the domains of *Prrx1* expression in the ventral regions of the mandibular and hyoid arches are similar to the expression domains of *Hand2*. Single *Prrx1* knockout mice display extensive malformations of the viscerocranium derived from the mandibular and hyoid arches, including fusion of the incus to palatoquadratum and fusion of the stapes to Reichert’s cartilage [191]. Single *Prrx2* knockout does not result in any discernible abnormalities in the craniofacial skeleton, suggesting that *Prrx1* compensates for the loss of *Prrx2*. However, compound *Prrx1* and *Prrx2* knockout mice have amplification of the craniofacial phenotype found in single *Prrx1* mutants [192]. As a result, the lower jaw is micrognathic, fused at its anterior tip and often has only a single incisor in the midline. The cause of this defect may have several explanations [193]. Firstly, the downregulation of *Shh* in the oral epithelium of compound *Prrx1*/*Prrx2* mutants can lead to reduced cell proliferation, resulting in the mandibular hypoplasia. Secondly, *Prrx1*/*Prrx2* double mutants contain a large population of *Runx2*-positive cells in the middle and rostral region, indicating precocious or accelerated osteogenesis in the mandibular process. Only remnants of Meckel’s cartilage in the rostral region of the mandibular process are preserved in *Prrx1/Prrx2* double mutants. Loss of the main body of Meckel’s cartilage and increased osteogenesis may be related to changes in the mesenchymal precursors from chondrogenic to osteogenic fate. Thirdly, the expanded domain of *Runx2* expression in mutants may be a result of increased proliferation or decreased apoptosis of osteogenic mesenchyme and/or the recruitment of additional osteoprogenitor cells to compensate for the loss of Meckel’s cartilage. Unlikely, but still possible, the expanded domain of expression of *Runx2* may reflect the fusion of multiple osteogenic condensations. Similar to mice, the homozygous or dominant heterozygous loss of *PRRX1* in humans has been associated with loss of the lower jaw [194,195,196,197,198]. *Prrx1/Prrx2* mouse mutants also display abnormalities in the dorsal hyoid arch. The stylohyoid ligament is not formed completely in the *Prrx1*-null mutant, and a part of it develops as a cartilaginous element [191]. In contrast, in compound *Prrx1* and *Prrx2* mutants, the entire stylohyoid ligament chondrifies and forms a continuous structure stretching from the stapes and styloid process to the lesser horns of the hyoid bone. Moreover, the chondrification of the stylohyoid ligament also occurs in *Prrx1^+/−^ Prrx2^−/−^* mice [92]. The accelerated osteogenesis in the *Prrx1/Prrx2* double mutants results in similar abnormalities as those in the *Hand* compound mutants and abnormalities in mice with the deletion of PA-specific enhancer *dHand* (*Hand2*), which also exhibited accelerated osteoblast differentiation [122,123]. Of note, the expanded *Dlx5*- and *Runx2*-positive domains and downregulation of *Shh* in the medial and oral region of the mandibular process are also found in *Meis2* NC-specific mutants [79]. The second PA phenotype of *Prrx1*/*Prrx2* double null mice is astoundingly similar to the hyoid abnormalities in *Meis2*-null mice, which suggests that there might be a link between *Prrx1*/*Prrx2* and *Meis2* during the hyoid arch development [92]

During the pharyngula stage, *Shh* influences the development of Meckel’s cartilage. Tissue-specific inactivation of *Shh* in the oropharyngeal epithelium in *Nkx2.1-Cre; Shh^flox^* leads to a complete lack of Meckel’s cartilage formation in the mouse embryos. In contrast, when SHH responsiveness is deleted from the ectomesenchyme using W*nt1-Cre2; Smo^flox^*, Meckel’s cartilage still develops, albeit truncated. Either SHH acts through an SMO-independent mechanism or its effect on Meckel’s cartilage is indirect through another signalling molecule, such as FGF8. 

Another member of the Hedgehog family, Indian Hedgehog (IHH), is a signalling molecule widely recognized as a regulator of skeletal development that is expressed in the prehypetrophic chondrocytes and early hypertrophic chondrocytes. In craniofacial morphogenesis, the expression of *Ihh* has traditionally been associated with the secondary cartilage—the mandibular symphysis, angular, coronoid, and condylar processes. In *Ihh*-null mice, the development of mandibular symphysis is defective due to precocious chondrocyte maturation and reduced proliferation of the chondroblast progenitors. However, this phenotype can be rescued upon ablation of *Gli3*, which thus acts as a negative regulator of symphyseal development [199,200]. Furthermore, IHH signalling during embryogenesis promotes the expression of parathyroid hormone-related protein (*Pthrp*) at the apical end of the presumptive condylar cartilage, thereby increasing numbers of presumptive chondroprogenitor cells. In keeping with this, the articular disc and temporomandibular joint are absent in *Ihh*-null mice, and the condylar process directly opposes the glenoid fossa. Interestingly, the disc phenotype of *Ihh*-null mice is not rescued in the concurrent absence of *Gli3* [199]. Conversely, tissue-specific augmentation of *Ihh* expression in the NCCs leads to severe craniofacial abnormalities, including a complete loss of the glenoid fossa [201]. In mice, IHH signalling via PTC1 controls the proliferation and differentiation of mesenchymal cells into chondrocytes during growth of the mesial alveolar process of dentary bone. Furthermore, *Ihh*-null newborn mice have the overall length of the mandibular body reduced by as much as a third, including secondary cartilages [202]. Likewise, in humans, patients carrying a mutation in *GLI2* exhibit a range of facial defects, including mandibular hypoplasia [203]. To summarize, IHH regulates a myriad of processes during craniofacial morphogenesis—including the proliferation and maturation rate of chondrocytes, endochondral ossification, the expression of *Pthrp* in periarticular tissue, articular disc formation, and synovial cavity formation.

Transforming growth factor beta (TGF-β) is a protein ligand that acts as a stimulant in cranial NCCs, increasing the proliferation of chondrocytes and the production of cartilage extracellular matrix [204]. Tissue-specific loss of *Tgfbr1* in NCCs causes delayed tooth initiation and mandibular defects, particularly in the proximal region, including loss of secondary mandibular cartilages [205]. Moreover, *Tgfbr1* mutants also exhibit a lack of the stapes and severe malformations of second PA cartilage with differentiation of multiple novel ectopic elements derived from the NC. Viscerocranial phenotypes of NC-specific *Tgfbr2* mouse mutants are less severe but comparable to those in *Tgfbr1* mutants [205,206]. Tissue-specific deletion of *Tgfbr2* in NCCs particularly affects the lower jaw and palate [207,208,209]. Explicitly, the elimination of *Tgfbr2* causes micrognathia and loss of secondary mandibular cartilages.

Signalling proteins from the BMP subfamily are major factors influencing the development of dentary bone, as ectopic expression of *Bmp* on the oral side of the mandibular process results in the formation of mirror-image dentary bone. The absence of two dedicated BMP4 antagonists, Chordin and Noggin, in the distal mandibular epithelium during the mandibular process patterning results in elevated levels of BMP4 at the expense of FGF8, increasing cell death and leading to the spectrum of mandibular hypoplasia, culminating in almost total agnathia [210]. In contrast, the absence of a single allele of BMP4 antagonist Noggin leads to significantly thicker cartilage along with increased pSmad1/5/8 expression, leading to ossification rather than degeneration of Meckel’s cartilage [211]. Similarly, in mutant mice with a complete lack of Noggin, the hyoid body is greatly enlarged, and its shape changes due to significantly shorter and wider horns [212]. Ectopic *Bmp4* expression in NCCs leads to bony fusion of the dentary and maxilla, which is reminiscent of the syngnathia birth defect in humans [213]. Upon ablation of *Bmp2* specifically in NCCs, the expression of *Sox9* is downregulated in both the mandibular process and Meckel’s cartilage, which results in micrognathia and cleft palate, characteristic features of Pierre Robin malformation sequence [214]. Compound loss of *Bmp2* and *Bmp4* in NCCs results in more severe shortening of mandible then in either of single mutants [215]. In mice with homozygous mutation in *Bmp5,* loss of the lesser horns and shortening of the greater horns of the hyoid occurs [216]. Mice with a deficiency of *Bmp7* exhibit shortening of maxilla and mandible, as well as a failure of Meckel’s cartilage fusion at the anterior tip [217]. 

Even though mutations in *Fgfs* and genes coding FGF receptors (FGFRs) are usually associated with craniosynostoses, these signalling molecules exert their influence over PAs as well. *Fgfr1* is expressed in the pharyngeal epithelium and is necessary to create a permissive environment for the immigration of NCCs. Mice homozygous for a hypomorphic allele of *Fgfr1* display reduced *Fgf3* expression in the pharyngeal epithelium, which prevents NCCs from entering the second PA and induces apoptosis instead [218]. In *Ffgr1* hypomorphs, craniofacial skeletal malformations occur mainly within the second PA and include a deficient or missing stapes and a missing proximal part of the styloid process. In general, the abnormalities in pharyngeal development in embryos in which FGF signalling has been disrupted are strikingly similar to each other, with the most consistent phenotypic feature being the hyoid arch hypoplasia [218,219,220,221,222]. Elimination of *Fgfr1* specifically in NCCs causes orofacial dysformation, tooth bud defects, and micrognathia [223]. One diagnostic feature of craniosynostosis syndromes in humans is mandibular dysgenesis. Gain-of-function mutation in *Fgfr2* leads to *Ffgr2*-related craniosynostosis and mandibular dysmorphogenesis, demonstrating that *Fgfr2* influences cartilage and intramembranous bone formation [224]. Additionally, in a mouse model of achondroplasia, gain-of-function mutation in *Fgfr3* leads to structural anomalies of Meckel’s cartilage and secondary mandibular cartilages, resulting in mandibular hypoplasia and dysmorphogenesis [225]. The formation of Meckel’s cartilage is critically dependent on the FGF8 molecule. Exogenous FGF8 is able to rescue Meckel’s cartilage in mouse mandibular explant cultures treated with the antagonist of HH signalling [226]. In the chick mandibular process, implanting SHH-soaked beads into the tissue leads to the development of supernumerary Meckel’s cartilage and an ectopic expression of *Fgf8*. Conversely, a significant reduction of *Fgf8* in the proximal region of the mandibular process is seen in mouse mutants with tissue-specific ablation of *Shh* in the oropharyngeal epithelium, which lack Meckel’s cartilage altogether [146]. Intriguingly, overexpression of *Fgf8* in NCCs leads to severe craniofacial malformations, including exencephaly, maxilla, and dentary bone agenesis [227]. In *Fgf8* hypomorphic mutants, both the mandibular and hyoid arches are obviously smaller, showing a reduction in total size. Moreover, these mutants have either absent or severely hypoplastic Meckel’s cartilage, absent malleus and incus; severely defective dentary bone and tympanic ring; and reduced or absent alisphenoid, presphenoid, squamous temporal bone, pterygoid, palatine, and ala temporalis bone. In *Fgf8* hypomorphic embryos, the stapes is normal or slightly smaller, the styloid process is thickened and/or shortened, and in a subset of mutants, the hyoid bone is mildly defective, which is surprising given the severity of defects noted in the early hyoid arch development [228]. *Fgf10* modulates the early morphogenesis of Meckel’s cartilage by controlling cell differentiation in the mandibular process. Overexpression of *Fgf10* in rat mandibular explants results in deformation of Meckel’s cartilage and a significant increase in its size, whilst also inducing the upregulation of cartilage specific genes, such as *Col2a1* and *Sox9* [229]. Accordingly, genetic polymorphism in *FGF10* has been linked with mandibular prognathism in humans [230]. The FGF pathway is overreactive in mice with mutations of *Sprouty* genes (*Spry*), which encode for inhibitors of receptor tyrosine kinases that are crucial for regulation of FGF downstream signalling. NC-specific deletion of *Spry1* results in malformed and incomplete maxilla, as well as a smaller mandible. Single *Spry4* loss or simultaneous loss of both *Spry2* and *Spry4* leads to micrognathia and growth retardation of the mandible, as well as incisor anomalies [231]. In summary, the combined action of the TGB-β, BMP, and FGF signalling pathways collectively controls the proliferation, maintenance, and cell fate specification of ectomesenchymal cells during osteogenic and chondrogenic differentiation. Furthermore, several mouse mutants with anomalies in the mandibular and hyoid arches have supernumerary pharyngeal skeletal elements, indicating that the ectomesenchyme in the head indeed retains an ability to form a spectrum of novel structures in response to either loss of cell signalling or ectopic cell signalling. These novel structures may represent skeletal atavisms and could be caused by a reactivation of a dormant developmental programme.

### 7.2. Zebrafish

Similar to the mouse, Wnt signalling is important for chondrogenic cell fate during craniofacial development in the zebrafish. The Wnt–Frizzled (Frz) complex modulates the jaw and ethmoid plate development [232]. Knockdown of *frzb* and *frzd7a* in zebrafish morphants results in lack of the lower jaw and ceratobranchial cartilage, as well as the loss of chondrocytes within the ethmoid plate. Altogether, the interaction between Wnt9a/Frzb/Frzd7a is crucial for the chondrogenic proliferation and cell fate within the ethmoid plate. Moreover, Wnt9/Frzb/Frzd7 also drive the development of the lower jaw. Ergo, Wnt–Frz complex regulates both canonical and planar-cell-polarity pathways during craniofacial chondrogenesis [233].

Wnt9a drives the expression of orthologue *sox9a* during early chondrogenic differentiation. Zebrafish *sox9a* mutants lack almost the entire set of cranial cartilage, except for the ceratohyal cartilage, which eventually leads to a reduction in a number of cranial intramembranous bones, including the dentary bone, maxilla, and the opercle [234,235]. On the other hand, *sox9b* knockdowns reveal a mild reduction of cartilages within mandibular and hyoid arches of morphants, which is in striking comparison with *sox9a* mutants [235]. Furthermore, *sox9b* knockdown mutants lack nearly all cranial bones, including most intramembranous bones, except for the cleithrum and the opercle [235]. However, the craniofacial phenotype remains unaffected in *sox9b*-null mutants [234]. All in all, analysis of *sox9a* zebrafish knockout mutants shows that *sox9a* is important during chondrogenic differentiation [235,236].

Two *runx2* orthologues, *runx2a* and *runx2b*, can be found within the zebrafish genome [236]. *Runx2b* is expressed throughout the mesenchyme of presumptive pharyngeal cartilages even before chondrogenic differentiation [236,237]. In contrast to murine *Runx2*, *runx2b* in zebrafish is expressed in all chondrocytes and is downregulated in *sox9b* mutants but remains unaffected in *sox9a* mutants [235]. *Runx2a* is expressed predominantly in the mandibular arch, while being expressed only weakly in the hyoid cartilages [236,237]. While *runx2b* morphants lack all pharyngeal cartilage, *runx2a* knockdown has barely any effect on pharyngeal chondrogenesis. Indeed, *runx2b* can possibly compensate for the lack of *runx2a* during pharyngeal skeletogenesis [237]. In contrast with mice, the expression of *runx2a*/*runx2b* is not affected by canonical Wnt and Fgf signalling during the early osteogenic differentiation [238]. Rather, Runx2b is induced by Runx3 emanating from the endoderm [237]. Following the induction of Runx3, Egr1 is activated in the endoderm, which in turn downregulates the expression of *sox9b* and *follistatin A* (*fsta*). Together, Runx3/Egr1/Sox9b/Fsta enable Bmp signalling during cranial cartilage development via inhibition of Bmp antagonists [239]. In addition, both Sox9 and Runx2 are affected by Foxe1 during osteochondrogenesis. Foxe1 suppresses Fgfr2, which in turn enables development of the cartilage within PAs [240]. Taken together, Wnt signalling and SOX9/Sox9a transcription factors are master regulators of chondrogenesis in both mice and zebrafish. Moreover, *Runx2* participates in chondrogenesis in both species. However, the key difference is that cartilage within PAs cannot form without *runx2b* in zebrafish, whilst in mice, it can form even when the expression of *Runx2* is lost. 

In contrast with *runx2*, *sp7* expression in zebrafish is regulated by canonical Wnt signalling, which acts in concert with Fgf to modulate osteogenic differentiation [238]. Further support for Wnt-mediated control of osteogenesis during craniofacial development in zebrafish can be found within developing intramembranous bones, which express a mediator of canonical Wnt signalling, *tcf7* [241]. Delayed ossification of maxilla occurs in *sp7* mutants, while other cranial intramembranous bones (cleithrum, brachiostegal rays, opercle, parasphenoid) are misshapen. In general, intramembranous bones of *sp7* mutants show reduced ossification, whereas the cartilage development appears unchanged, as the expression of chondrocyte differentiation markers *sox9a*, *sox9b*, *runx2a*, and *runx2b* is unaltered. However, the expression of osteogenesis-related markers, such as *bglap*, *spp1*, *col1a1a*, and *col1a1b*, is decreased. Of note, *sp7* is a driver of *col10a1a* expression in osteoblasts, and the role of *col10a1a* during osteoblastogenesis has only been described in zebrafish so far [241,242,243]. 

In the zebrafish neurula, Shh signalling radiates from the ventral brain primordium into the presumptive stomodeal area. When NCCs colonize the mandibular arch, a signal from the stomodeum diffuses into anterior NCCs, initiating the mesenchymal condensation and giving rise to the pterygoid process of palatoquadrate cartilage and the neurocranium. Double knockdown of *shh* and *twhh*, two members of the *hh* family, leads to the loss of ectomesenchymal condensations and the prospective anterior cranial skeleton [244]. In addition, Hh signalling influences development of the jaw joint via regulation of *nkx3.2* and *gd5* [245].

Later in the craniofacial development, Hh signalling via Ptc1 and Ptc2 plays a role during the differentiation of osteoblasts in the perichondrium of ceratohyal and hyosymplectic cartilages [246]. In *ihha*-null zebrafish, reduced proliferation of chondrocytes is reflected in the loss of mineralized endochondral bones in the cranium. Depending on the concentration, *ihha* regulates chondrogenic and osteogenic proliferation via Gli1 and Gli3 transcription factors [247]. In addition, Hh signalling drives the proliferation of preosteoblasts during the intramembranous osteogenesis [248]. During the endochondral bone formation, Hh signalling influences the expression of *runx2a*, *runx2b*, *sp7*, and *colX* in ossification centres [246]. Not unlike *ihh*, s*hh* also plays a role during the ossification and mineralization, as it upregulates the expression of *bmp2*, *sp7*, and *col10a1a*. Moreover, Hh signalling has also been discovered to downregulate autophagy during osteoblastogenesis [249].

In contrast with mice, Meckel’s cartilage is affected only mildly in zebrafish *prrx1a/prrx1b* mutants, whereas dorsal pharyngeal cartilages are affected more significantly. During the chondrogenic differentiation, *prrx1a*/*prrx1b* are negatively regulated by Edn1 in ventral pharyngeal cartilages. Conversely, Jag1b-Notch signalling in concert with Prrx1a/Prrx1b sets up dorsal pharyngeal cartilages via inhibition of *barx1* [250]. Intriguingly, both zebrafish and mice exhibit ectopic and abnormal cartilage in the dorsal region of mandibular and hyoid arches; however, loss of *prrx1a* and *prrx1b* in the zebrafish produces very minor defects in ventral cartilages. In contrast, knockout of *Prrx1* in mice produces extensive malformations within the mandibular process and both ventral and dorsal hyoid arches. 

Knockdown of *tgfbr2* in zebrafish causes the shortening of jaws and misshapen palate [251], which is similar to findings in mouse mutants. In the zebrafish, *tgfβ-3* governs the generation and survival of the cranial NC from premigratory to migratory stages, and its inhibition leads to the increased apoptosis of NCCs, resulting in malformations of the palatoquadrate and other cranial cartilages [252]. Zebrafish *fgfr3* knockout mutants display malformations of the mandible and delayed ossification of the craniofacial skeleton. Both early stage osteoblast markers, such as *col10a1a*, and late-stage osteoblast markers, such as *spp1, osn*, and *col1a2*, exhibit decreased expression. Additionally, upregulation of the chondrogenic proliferation and irregular directional orientation of chondrocytes can be observed in *fgfr3* mutant fish, accompanied by an upregulation of Ihh and canonical Wnt signalling [3]. Thus, *fgfr3* downregulates Wnt/ß-catenin and Ihh signalling. The importance of Fgf signalling during zebrafish chondrogenesis is further supported by knockdown experiments of *fgf10a*, which cause the reduction of Meckel’s cartilage and deformation of the palatoquadrate cartilage [251]. Bmp is another signalling pathway that is crucial for ossification of the cranial skeleton in the zebrafish. It is mediated via the production of nitric oxide [253] Knockdown of zebrafish orthologues *msxB*, *msxC*, and *msxE* leads to the loss of jaws, including other ventral pharyngeal cartilages, as a result of arch ventralization [254].

## 8. Conclusions

The pharyngeal apparatus is one of the hallmarks of gnathostome embryogenesis and evolution. The neural crest gives rise to most of the tissue within PA-derived skeletal elements, which collectively form the viscerocranium. The expression of *Hox* genes in the hindbrain and NCCs specifies the identity of individual PAs. Cell identity in the mandibular arch is regulated by the MEIS/PBX complex, whereas in the hyoid arch, a trimeric complex of HOX/MEIS/PBX specifies the second PA fate. The skeletal polarity within the individual PA is governed by the EDN–DLX–HAND regulatory cascade. Numerous signalling pathways operating within PAs, including FGF, BMP, and SHH, pattern the nascent arch and thus ensure the genesis of heterogenous structures, including teeth, skeletal components, and the tongue. In this review, we have discussed the role of transcription factors SOX9, RUNX2, SP7, PAX3, GSC, MSX, and PRRX and the signalling pathways WNT and HH during mandibular and hyoid arch skeletogenesis. Within the PAs, these molecules influence chondrogenic and osteoblastic differentiation, the transition from chondroblastic to osteoblastic fate, and the choice between osteoblastic and chondroblastic cell fate. In addition, multiple ligands from the TGF-β, BMP, and FGF families control the proliferation, maintenance, and differentiation of NCCs in conjunction with the aforementioned transcription factors. Disruption of these signalling pathways in the ectomesenchyme within the mandibular and hyoid arches results in malformations and dysmorphies of the viscerocranium.

In contrast with zebrafish, the hyoid arch is rarely a subject of interest in mice and humans. Certainly, one reason for this is a scarcity of patients with hyoid arch abnormalities in a clinical setting. As the mandibular arch has a much larger impact on development of the viscerocranium than the hyoid arch, morphogenesis of the hyoid arch has naturally been less studied by researchers. Nonetheless, malformations in the mandibular arch often co-occur with hyoid arch malformations in mouse genetic mutants. Although hyoid abnormalities have not been casually reported in human patients with first arch syndromes, they have been described to co-occur with Pierre Robins sequence, 22q11.2 deletion syndrome, and cleft lip/cleft palate, which highlights the importance of the management of hyoid abnormalities in patients with first arch anomalies. In a clinical setting, symptomatic anatomical variants of the hyoid–larynx complex can often be overlooked by physicians. Nonetheless, it seems plausible that the first arch anomalies are often accompanied with hyoid anomalies in humans but remain unnoticed or underreported. 

It is important to note that signalling molecules and transcription factors governing PA development are similar between the mandibular and hyoid arches, which is in fact already known from studies in zebrafish. In both mice and zebrafish, the *Edn1*, *Dlx5*/*Dlx6*, and *Hand2* genes control cell fate in the ventral regions of the mandibular and hyoid arches—the mandibular process and the ventral hyoid arch—and, accordingly, a mutation in any of them results in malformations particularly in the lower jaw and lesser horns of the hyoid. *Dlx1*/*Dlx2* specify dorsal regions of the mandibular and hyoid arches, and their absence does not result in malformations of the ventral PA derivatives. In mice, the expression of the *Meis* and *Pbx* genes in the mandibular and hyoid arches largely overlaps with *Hand2*, and both *Meis* and *Pbx* mutants display malformations of the mandibular process and the hyoid bone. Therefore, *Meis*, *Pbx*, *Hand2*, and *Prrx1* share similar temporospatial patterns of expression in PAs—weak expression in lateral regions, while being abundant in medial regions. This pattern of expression in medial domains of the mandibular and hyoid arches suggests a tight link between the medial structures of first two PAs. Although *meis* and *pbx* govern both mandibular and hyoid arches, the precise link between *pbx*/*meis* and *hand* is not well studied in zebrafish. In both zebrafish and mice, *Prrx1* is involved in the specification of the dorsal fate of the mandibular and hyoid arches, whereas in mice, *Prrx1* participates in the specification of both ventral and dorsal fates of the mandibular and hyoid arches. In conclusion, we presume that the development of mandibular and hyoid arches involves a common regulatory network involving MEIS, PBX, EDN1, DLX5/6, HAND2, and PRRX1 that is shared among gnathostomes.

## Figures and Tables

**Table 1:** Skeletal derivatives of PAs in the mouse and zebrafish.

Viscerocranium	Mouse		Zebrafish	
	Cartilaginous	Membranous	Cartilaginous	Membranous
First pharyngeal arch (the mandibular)	Palatoquadrate cartilage: Alisphenoid Incus	Premaxilla Maxilla Zygomatic bone Temporal squama	Palatoquadrate cartilage: Quadrate Metapterygoids Palatines	Premaxilla Maxilla Ectopterygoid Entopterygoid
	Meckel’s cartilage: Mandibular symphysis Lingula of mandible Sphenomandibular ligament Spine of sphenoid Anterior ligament of malleus Malleus	Dentary bone	Meckel’s cartilage: Retroarticular	Dentary Anguloarticular Coronomeckelian
Second pharyngeal arch (the hyoid)	Stapes Styloid process of the temporal bone Stylohyoid ligament Lesser horns of the hyoid bone		Basihyal Ceratohyal Epihyal Hypohyal Hyomandibula Interhyal Symplectic	Urohyals Branchiostegal rays Interopercle Opercles Preopercles Subopercles
Third pharyngeal arch	Greater horns of the hyoid bone		Basibranchials Ceratobranchials Epibranchials Hypobranchials Pharyngobranchials	

## Data Availability

Not applicable.
